# Supplementary material for: Drinking water and rural schools in the Western Amazon: an environmental intervention study
Source: PeerJ. 2018 Jun 15;6:e4993. doi: 10.7717/peerj.4993 (PMC6005166; doi:10.7717/peerj.4993)
Supplement: Supplemental Information 1 [file peerj-06-4993-s001.zip › Lab_Reports_after_Intervention.zip!/mpdf-ESCOLA MAJOR JOÃO CÂNCIO.pdf]

**Governo do Estado do Acre**
**Secretaria Estadual de Saúde**
**LABORATORIO CENTRAL DE SAUDE PUBLICA**
Travessa do Hemoacre, 165 - Vila Ivonete
CNPJ: 04.034.526/0021-97
**Resp. Técnico:** Isaias Santos de Mesquita - **CRF:** CRBM N° 2679
**Site:** http://www.lacen.ac.gov.br - **E-mail:** produtos.lacen@ac.gov.br
**Telefone:** (68)3228-5355 - **Fax:** (68)3228-2720

# RELATÓRIO DE ENSAIOS

## Nº170118000238

Nº Vigilância: 17051701

## DADOS DO SOLICITANTE

**Nome:** SECRETARIA MUNICIPAL DE SAUDE DE RIO BRANCO (CNES: 5889030)
**Município:** RIO BRANCO / AC
**Natureza:** PÚBLICA **Origem:** VIGIAGUA

## DADOS DA COLETA

**Finalidade:** VIGIAGUA MENSAL
**Motivo:** POTABILIDADE
**Local:** ESCOLA RURAL MAJOR JOAO CARCIO
**Endereço:** AC 90 KM 80 TRASACREANA
**Município:** RIO BRANCO / AC
**Zona:** RURAL
**Procedência da Coleta:** INTRA-DOMICILIAR/INTRA-PREDIAL
**Ponto da Coleta:** TORNEIRA APÓS A RESERVAÇÃO
**Responsável:** BARBOSA **Documento:** RG 0118787 **Telefone:** (68)9998-4150

## DADOS DA AMOSTRA

**Tipo da Amostra:** ÁGUA NÃO TRATADA **Apresentação:** 300 mL **Acondicionamento:** GELO RECICLADO
**Data da Coleta:** 16/05/2017 **Hora da Coleta:** 09h 40min **Chuva nas últimas 48hs:** SIM

## ANÁLISE DE CAMPO

**Cloro Residual Livre :** 0.00 mg/L **Fluor:** n mg/L **Temperatura:** n ºC **pH:** n **Turbidez:** n uT

## RECEBIMENTO DA AMOSTRA

**Data:** 17/05/2017 **Hora:** 08h 36min **Entregue por:** MAX **Recebido por:** ARLENE

## RESULTADO DAS ANÁLISES

## MICROBIOLÓGICA

**Ensaio:** COLIFORMES TOTAIS **Data Final Processamento:** 18/05/2017 09h 30min
**Referência:** PORTARIA Nº 2.914, DE 12 DE DEZEMBRO DE 2011 **Valor Ref.:** Ausência em 100 mL
**Metodologia:** Substrato Cromogênico/Enzimático SMEWW, 22ª Ed. 9223 B
**Resultado:** Presença
**Conclusão:** Não Se Aplica
*Conferido e liberado por **OLAVIO AUGUSTO DA SILVA NUNES(Médico Vet. CRMV/AC - 74)**, em 18/05/2017 09:28:28.*

---

**Ensaio:** ESCHERICHIA COLI **Data Final Processamento:** 18/05/2017 09h 30min
**Referência:** PORTARIA Nº 2.914, DE 12 DE DEZEMBRO DE 2011 **Valor Ref.:** Ausência em 100 mL
**Metodologia:** Substrato Cromogênico/Enzimático SMEWW, 22ª Ed. 9223 B
**Resultado:** Ausência
**Conclusão:** Satisfatório
*Conferido e liberado por **OLAVIO AUGUSTO DA SILVA NUNES(Médico Vet. CRMV/AC - 74)**, em 18/05/2017 09:28:27.*

---

# RELATÓRIO DE ENSAIOS

## Nº170118000238

Nº Vigilância: 17051701

## ORGANOLÉPTICA

**Ensaio:** TURBIDEZ **Data Final Processamento:** 17/05/2017 15h 36min
**Referência:** PORTARIA Nº 2.914, DE 12 DE DEZEMBRO DE 2011 **Valor Ref.:** VMP: 5 uT
**Metodologia:** Método Nefelométrico SMEWW, 22ª Ed. 2130 B
**Resultado:** 4,95 uT
**Conclusão:** Satisfatório

*Conferido e liberado por* ***MARCIA SOCORRO MENDES DA SILVA(Biomédica CRBM 4ª - 776/PA)****, em 17/05/2017 16:01:22.*

## CONCLUSÃO FINAL

***SATISFATÓRIA***

Satisfatório para os ensaios realizados.

*Conferido e liberado por* ***OLAVIO AUGUSTO DA SILVA NUNES(Médico Vet. CRMV/AC - 74)****, em 18/05/2017 09:29:27.*

**Notas:**
**1 -** VMP: Valor Máximo Permitido | VR: Valor de Referência;
**2 -** LQM: Limite de Quantificação do Método | LDM: Limite de Detecção do Método;
**3 -** SAA: Sistema de Abastecimento de Água | SAC: Solução Alternativa Coletiva | SAI: Solução Alternativa Individual;
**4 -** SMEWW: Standard Methods for the Examination of Water & Wastewater | APHA: American Public Health Association | NBR: Norma Brasileira;
**5 -** São de responsabilidade do solicitante o plano amostral, os dados da coleta, a coleta, o acondicionamento, o transporte e análise de campo;
**6 -** O relatório não pode ser utilizado em publicidade, propaganda e/ou para fins comerciais. Os resultados referem-se única e exclusivamente à amostra encaminhada pelo solicitante;
**7 -** Considerar Ausência em 100mL como valor de referência para Coliformes Totais apenas para amostra de ETA coletada na saída do tratamento/pós desinfecção. Nos outros pontos de coleta não há valor de referência para Coliformes Totais..
