# Supplementary material for: Drinking water and rural schools in the Western Amazon: an environmental intervention study
Source: PeerJ. 2018 Jun 15;6:e4993. doi: 10.7717/peerj.4993 (PMC6005166; doi:10.7717/peerj.4993)
Supplement: Supplemental Information 1 [file peerj-06-4993-s001.zip › Lab_Reports_after_Intervention.zip!/mpdf-ESCOLA RAIMUNDO HERMÍNIO DE MELO.pdf]

Governo do Estado do Acre
Secretaria Estadual de Saúde
LABORATORIO CENTRAL DE SAUDE PUBLICA
Travessa do Hemoacre, 165 - Vila Ivonete
CNPJ: 04.034.526/0021-97
Resp. Técnico: Isaias Santos de Mesquita - **CRF:** CRBM N° 2679
Site: http://www.lacen.ac.gov.br - **E-mail:** produtos.lacen@ac.gov.br
Telefone: (68)3228-5355 - **Fax:** (68)3228-2720

RELATÓRIO DE ENSAIOS

Nº170118000327

Nº Vigilância: 17053101

DADOS DO SOLICITANTE

Nome: SECRETARIA MUNICIPAL DE SAUDE DE RIO BRANCO (CNES: 5889030)
Município: RIO BRANCO / AC
Telefone: (68)3227-3165
Natureza: PÚBLICA **Origem:** VIGIAGUA

DADOS DA COLETA

Finalidade: VIGIAGUA MENSAL
Motivo: POTABILIDADE
Local: ESCOLA RURAL RAIMUNDO HERMÍNIO DE MELO
Endereço: RUA MILTON MACIEL S/N, SANTA CECÍLIA
Município: RIO BRANCO / AC
Zona: RURAL
Procedência da Coleta: INTRA-DOMICILIAR/INTRA-PREDIAL
Ponto da Coleta: TORNEIRA APÓS A RESERVAÇÃO
Responsável: MAX **Documento:** RG 145422 **Telefone:** (68)9952-9315

DADOS DA AMOSTRA

Tipo da Amostra: ÁGUA TRATADA **Apresentação:** 300 mL **Acondicionamento:** GELO RECICLADO
Data da Coleta: 31/05/2017 **Hora da Coleta:** 08h 45min **Chuva nas últimas 48hs:** SIM

ANÁLISE DE CAMPO

Cloro Residual Livre : 0,34 mg/L **Fluor:** N mg/L **Temperatura:** N ºC **pH:** N **Turbidez:** N uT

RECEBIMENTO DA AMOSTRA

Data: 31/05/2017 **Hora:** 16h 35min **Entregue por:** BARBOSA **Recebido por:** SOCORRO SILVA

RESULTADO DAS ANÁLISES

MICROBIOLÓGICA

Ensaio: COLIFORMES TOTAIS **Data Final Processamento:** 01/06/2017 16h 55min
Referência: PORTARIA Nº 2.914, DE 12 DE DEZEMBRO DE 2011 **Valor Ref.:** Ausência em 100 mL
Metodologia: Substrato Cromogênico/Enzimático SMEWW, 22ª Ed. 9223 B
Resultado: Ausência
Conclusão: Não Se Aplica
Observação: Considerar Ausência em 100mL como valor de referência apenas para amostra coletada na saída do tratamento. Nos outros pontos de coleta não há valor de referência para Coliformes Totais.

*Conferido e liberado por **FRANCISCO CARLOS DE ABREU MENEZES(Biomédico CRBM-4N°780-PA)**, em 05/06/2017 14:50:23.*

Ensaio: ESCHERICHIA COLI **Data Final Processamento:** 01/06/2017 16h 55min
Referência: PORTARIA Nº 2.914, DE 12 DE DEZEMBRO DE 2011 **Valor Ref.:** Ausência em 100 mL
Metodologia: Substrato Cromogênico/Enzimático SMEWW, 22ª Ed. 9223 B
Resultado: Ausência
Conclusão: Satisfatório
*Conferido e liberado por **FRANCISCO CARLOS DE ABREU MENEZES(Biomédico CRBM-4N°780-PA)**, em 05/06/2017 14:50:23.*

Governo do Estado do Acre
Secretaria Estadual de Saúde
LABORATORIO CENTRAL DE SAUDE PUBLICA
Travessa do Hemoacre, 165 - Vila Ivonete
CNPJ: 04.034.526/0021-97
Resp. Técnico: Isaias Santos de Mesquita - **CRF:** CRBM N° 2679
Site: http://www.lacen.ac.gov.br - **E-mail:** produtos.lacen@ac.gov.br
Telefone: (68)3228-5355 - **Fax:** (68)3228-2720

RELATÓRIO DE ENSAIOS

Nº170118000327

Nº Vigilância: 17053101

ORGANOLÉPTICA

Ensaio: TURBIDEZ **Data Final Processamento:** 31/05/2017 16h 52min
Referência: PORTARIA Nº 2.914, DE 12 DE DEZEMBRO DE 2011 **Valor Ref.:** VMP: 5 uT
Metodologia: Método Nefelométrico SMEWW, 22ª Ed. 2130 B
Resultado: 0,72 uT
Conclusão: Satisfatório

Conferido e liberado por ***SANDRA MARIA MARTINS DE SOUZA(Quimica CRQ 14100456)****, em 06/06/2017 16:17:32.*

CONCLUSÃO FINAL

SATISFATÓRIA

Satisfatório em relação ao(s) ensaio(s) realizado(s).
O(s) resultado(s) deste laudo referem-se única e exclusivamente à amostra encaminhada pelo solicitante.

Conferido e liberado por ***SANDRA MARIA MARTINS DE SOUZA(Quimica CRQ 14100456)****, em 06/06/2017 16:21:51.*

Notas:
1 - VMP: Valor Máximo Permitido | VR: Valor de Referência;
2 - LQM: Limite de Quantificação do Método | LDM: Limite de Detecção do Método;
3 - SAA: Sistema de Abastecimento de Água | SAC: Solução Alternativa Coletiva | SAI: Solução Alternativa Individual;
4 - SMEWW: Standard Methods for the Examination of Water & Wastewater | APHA: American Public Health Association | NBR: Norma Brasileira;
5 - São de responsabilidade do solicitante o plano amostral, os dados da coleta, a coleta, o acondicionamento, o transporte e análise de campo;
6 - O relatório não pode ser utilizado em publicidade, propaganda e/ou para fins comerciais. Os resultados referem-se única e exclusivamente à amostra encaminhada pelo solicitante;
7 - Considerar Ausência em 100mL como valor de referência para Coliformes Totais apenas para amostra de ETA coletada na saída do tratamento/pós desinfecção. Nos outros pontos de coleta não há valor de referência para Coliformes Totais..
