# Supplementary material for: Drinking water and rural schools in the Western Amazon: an environmental intervention study
Source: PeerJ. 2018 Jun 15;6:e4993. doi: 10.7717/peerj.4993 (PMC6005166; doi:10.7717/peerj.4993)
Supplement: Supplemental Information 1 [file peerj-06-4993-s001.zip › Lab_Reports_after_Intervention.zip!/mpdf-ESCOLA ZAQUEU MACHADO.pdf]

Governo do Estado do Acre
Secretaria Estadual de Saúde
LABORATORIO CENTRAL DE SAUDE PUBLICA
Travessa do Hemoacre, 165 - Vila Ivonete
CNPJ: 04.034.526/0021-97
Resp. Técnico: Isaias Santos de Mesquita - **CRF:** CRBM N° 2679
Site: http://www.lacen.ac.gov.br - **E-mail:** produtos.lacen@ac.gov.br
Telefone: (68)3228-5355 - **Fax:** (68)3228-2720

RELATÓRIO DE ENSAIOS

Nº170118000527

Nº Vigilância: 17071022

DADOS DO SOLICITANTE

Nome: SECRETARIA MUNICIPAL DE SAUDE DE RIO BRANCO (CNES: 5889030)
Município: RIO BRANCO / AC
Telefone: (68)3227-3165
Natureza: PÚBLICA **Origem:** VIGIAGUA

DADOS DA COLETA

Finalidade: VIGIAGUA MENSAL
Motivo: POTABILIDADE
Local: ESCOLA ZAQUEU MACHADO
Endereço: BR 317 RAMAL MEDITERRANEO, KM 07, BAIXA VERDE
Município: RIO BRANCO / AC
Zona: RURAL
Procedência da Coleta: INTRA-DOMICILIAR/INTRA-PREDIAL
Ponto da Coleta: TORNEIRA APÓS A RESERVAÇÃO
Responsável: LUCIA **Documento:** RG 361899 **Telefone:** (68)9945-2816

DADOS DA AMOSTRA

Tipo da Amostra: ÁGUA TRATADA **Apresentação:** 300 mL **Acondicionamento:** GELO RECICLADO
Data da Coleta: 10/07/2017 **Hora da Coleta:** 10h 15min **Chuva nas últimas 48hs:** NÃO

ANÁLISE DE CAMPO

Cloro Residual Livre : 0,31 mg/L **Fluor:** n mg/L **Temperatura:** n ºC **pH:** n **Turbidez:** n uT

RECEBIMENTO DA AMOSTRA

Data: 11/07/2017 **Hora:** 07h 50min **Entregue por:** MAX **Recebido por:** ARLENE

RESULTADO DAS ANÁLISES

MICROBIOLÓGICA

Ensaio: COLIFORMES TOTAIS **Data Final Processamento:** 13/07/2017 07h 47min
Referência: PORTARIA Nº 2.914, DE 12 DE DEZEMBRO DE 2011 **Valor Ref.:** Ausência em 100 mL
Metodologia: Substrato Cromogênico/Enzimático SMEWW, 22ª Ed. 9223 B
Resultado: Ausência
Conclusão: Não Se Aplica
*Conferido e liberado por **OLAVIO AUGUSTO DA SILVA NUNES(Médico Vet. CRMV/AC - 74)**, em 13/07/2017 07:53:29.*

Ensaio: ESCHERICHIA COLI **Data Final Processamento:** 13/07/2017 07h 47min
Referência: PORTARIA Nº 2.914, DE 12 DE DEZEMBRO DE 2011 **Valor Ref.:** Ausência em 100 mL
Metodologia: Substrato Cromogênico/Enzimático SMEWW, 22ª Ed. 9223 B
Resultado: Ausência
Conclusão: Satisfatório
*Conferido e liberado por **OLAVIO AUGUSTO DA SILVA NUNES(Médico Vet. CRMV/AC - 74)**, em 13/07/2017 07:53:29.*

Governo do Estado do Acre
Secretaria Estadual de Saúde
LABORATORIO CENTRAL DE SAUDE PUBLICA
Travessa do Hemoacre, 165 - Vila Ivonete
CNPJ: 04.034.526/0021-97
Resp. Técnico: Isaias Santos de Mesquita - **CRF:** CRBM N° 2679
Site: http://www.lacen.ac.gov.br - **E-mail:** produtos.lacen@ac.gov.br
Telefone: (68)3228-5355 - **Fax:** (68)3228-2720

RELATÓRIO DE ENSAIOS

Nº170118000527

Nº Vigilância: 17071022

ORGANOLÉPTICA

Ensaio: TURBIDEZ **Data Final Processamento:** 11/07/2017 15h 40min
Referência: PORTARIA Nº 2.914, DE 12 DE DEZEMBRO DE 2011 **Valor Ref.:** VMP: 5 uT
Metodologia: Método Nefelométrico SMEWW, 22ª Ed. 2130 B
Resultado: 1,79 uT
Conclusão: Satisfatório
*Conferido e liberado por **MARCIA SOCORRO MENDES DA SILVA(Biomédica CRBM 4ª - 776/PA)**, em 11/07/2017 16:39:32.*

CONCLUSÃO FINAL

SATISFATÓRIA
Satisfatório para os ensaios realizados.
*Conferido e liberado por **OLAVIO AUGUSTO DA SILVA NUNES(Médico Vet. CRMV/AC - 74)**, em 13/07/2017 09:14:04.*

Notas:
1 - VMP: Valor Máximo Permitido | VR: Valor de Referência;
2 - LQM: Limite de Quantificação do Método | LDM: Limite de Detecção do Método;
3 - SAA: Sistema de Abastecimento de Água | SAC: Solução Alternativa Coletiva | SAI: Solução Alternativa Individual;
4 - SMEWW: Standard Methods for the Examination of Water & Wastewater | APHA: American Public Health Association | NBR: Norma Brasileira;
5 - São de responsabilidade do solicitante o plano amostral, os dados da coleta, a coleta, o acondicionamento, o transporte e análise de campo;
6 - O relatório não pode ser utilizado em publicidade, propaganda e/ou para fins comerciais. Os resultados referem-se única e exclusivamente à amostra encaminhada pelo solicitante;
7 - Considerar Ausência em 100mL como valor de referência para Coliformes Totais apenas para amostra de ETA coletada na saída do tratamento/pós desinfecção. Nos outros pontos de coleta não há valor de referência para Coliformes Totais..
