# Supplementary material for: Drinking water and rural schools in the Western Amazon: an environmental intervention study
Source: PeerJ. 2018 Jun 15;6:e4993. doi: 10.7717/peerj.4993 (PMC6005166; doi:10.7717/peerj.4993)
Supplement: Supplemental Information 1 [file peerj-06-4993-s001.zip › Lab_Reports_before_Intervention.zip!/mpdf-ESCOLA MAJOR JOÃO CÂNCIO.pdf]

Governo do Estado do Acre
Secretaria Estadual de Saúde
LABORATORIO CENTRAL DE SAUDE PUBLICA
Travessa do Hemoacre, 165 - Vila Ivonete
CNPJ: 04.034.526/0021-97
Resp. Técnico: Isaias Santos de Mesquita - **CRF:** CRBM N° 2679
Site: http://www.lacen.ac.gov.br - **E-mail:** produtos.lacen@ac.gov.br
Telefone: (68)3228-5355 - **Fax:** (68)3228-2720

RELATÓRIO DE ENSAIOS

Nº130118000818

Nº Vigilância: 13091812

DADOS DO SOLICITANTE

Nome: SECRETARIA MUNICIPAL DE SAUDE DE RIO BRANCO (CNES: 5889030)
Município: RIO BRANCO / AC
Telefone: (68)3227-3165 / **E-mail:** vigiaguasemsa@gmail.com
Natureza: PÚBLICA **Origem:** VIGIAGUA

DADOS DA COLETA

Finalidade: VIGIAGUA
Motivo: POTABILIDADE
Local: ESCOLA MAJOR JOÃO CÂNCIO
Endereço: RODOVIA AC 90 KM 82
Município: RIO BRANCO / AC
Zona: RURAL
Referência do Local da Coleta: ÁGUA PROVENIENTE DE UM AÇUDE ONDE ANIMAIS TOMAM BANHO
Procedência da Coleta: ESTABELECIMENTO DE ENSINO
Ponto da Coleta: TORNEIRA APÓS A RESERVAÇÃO
Informações Adicionais do Ponto da Coleta: ÁGUA DE AÇUDE
Responsável: LUCIA **Documento:** RG 361899 **Telefone:** (68)9998-4150

DADOS DA AMOSTRA

Tipo da Amostra: ÁGUA NÃO TRATADA **Apresentação:** 400 mL **Acondicionamento:** GELO RECICLADO
Data da Coleta: 18/09/2013 **Hora da Coleta:** 14h 35min **Chuva nas últimas 48hs:** SIM

ANÁLISE DE CAMPO

Não informado pelo responsável da coleta.

RECEBIMENTO DA AMOSTRA

Data: 19/09/2013 **Hora:** 09h 15min **Entregue por:** MAX **Recebido por:** ARLENE

RESULTADO DAS ANÁLISES

MICROBIOLÓGICA

Ensaio: COLIFORMES TOTAIS **Data Final Processamento:** 23/09/2013 08h 09min
Referência: PORTARIA Nº 2.914, DE 12 DE DEZEMBRO DE 2011 **Valor Ref.:**
Metodologia: Substrato Cromogênico/Enzimático SMEWW, 21ª Ed.9223B
Resultado: Presença
Conclusão: Não Se Aplica
*Conferido e liberado por **OLAVIO AUGUSTO DA SILVA NUNES(Médico Vet. CRMV/AC - 74)**, em 23/09/2013 08:14:12.*

Ensaio: ESCHERICHIA COLI **Data Final Processamento:** 23/09/2013 08h 10min
Referência: PORTARIA Nº 2.914, DE 12 DE DEZEMBRO DE 2011 **Valor Ref.:** Ausência em 100 mL
Metodologia: Substrato Cromogênico/Enzimático SMEWW, 21ª Ed.9223B
Resultado: Presença
Conclusão: Insatisfatório
*Conferido e liberado por **OLAVIO AUGUSTO DA SILVA NUNES(Médico Vet. CRMV/AC - 74)**, em 23/09/2013 08:14:12.*

Governo do Estado do Acre
Secretaria Estadual de Saúde
LABORATORIO CENTRAL DE SAUDE PUBLICA
Travessa do Hemoacre, 165 - Vila Ivonete
CNPJ: 04.034.526/0021-97
Resp. Técnico: Isaias Santos de Mesquita - **CRF:** CRBM N° 2679
Site: http://www.lacen.ac.gov.br - **E-mail:** produtos.lacen@ac.gov.br
Telefone: (68)3228-5355 - **Fax:** (68)3228-2720

RELATÓRIO DE ENSAIOS

Nº130118000818

Nº Vigilância: 13091812

ORGANOLÉPTICA

Ensaio: COR APARENTE **Data Final Processamento:** 19/09/2013 15h 34min
Referência: PORTARIA Nº 2.914, DE 12 DE DEZEMBRO DE 2011 **Valor Ref.:** VMP: 15 uH
Metodologia: Método Espectrofotométrico SMEWW, 21ª Ed. 2120C
Resultado: 138 uH
Conclusão: Insatisfatório
*Conferido e liberado por **SANDRA MARIA MARTINS DE SOUZA(Quimica CRQ 14100456)**, em 20/09/2013 12:45:31.*

Ensaio: TURBIDEZ **Data Final Processamento:** 19/09/2013 14h 55min
Referência: PORTARIA Nº 2.914, DE 12 DE DEZEMBRO DE 2011 **Valor Ref.:** VMP: 5 uT
Metodologia: Método Nefelométrico SMEWW, 21ª Ed. 2130B
Resultado: 16 uT
Conclusão: Insatisfatório
*Conferido e liberado por **SANDRA MARIA MARTINS DE SOUZA(Quimica CRQ 14100456)**, em 20/09/2013 12:45:31.*

CONCLUSÃO FINAL

INSATISFATÓRIA

*Conferido e liberado por **SANDRA MARIA MARTINS DE SOUZA(Quimica CRQ 14100456)**, em 23/09/2013 09:09:20.*

Notas:
1 - VMP: Valor Máximo Permitido | VR: Valor de Referência;
2 - LQM: Limite de Quantificação do Método | LDM: Limite de Detecção do Método;
3 - SAA: Sistema de Abastecimento de Água | SAC: Solução Alternativa Coletiva | SAI: Solução Alternativa Individual;
4 - SMEWW: Standard Methods for the Examination of Water & Wastewater | APHA: American Public Health Association | NBR: Norma Brasileira;
5 - São de responsabilidade do solicitante o plano amostral, os dados da coleta, a coleta, o acondicionamento, o transporte e análise de campo;
6 - O relatório não pode ser utilizado em publicidade, propaganda e/ou para fins comerciais. Os resultados referem-se única e exclusivamente à amostra encaminhada pelo solicitante;
7 - Considerar Ausência em 100mL como valor de referência para Coliformes Totais apenas para amostra de ETA coletada na saída do tratamento/pós desinfecção. Nos outros pontos de coleta não há valor de referência para Coliformes Totais..
