# Supplementary material for: Drinking water and rural schools in the Western Amazon: an environmental intervention study
Source: PeerJ. 2018 Jun 15;6:e4993. doi: 10.7717/peerj.4993 (PMC6005166; doi:10.7717/peerj.4993)
Supplement: Supplemental Information 1 [file peerj-06-4993-s001.zip › Lab_Reports_before_Intervention.zip!/mpdf-ESCOLA RAIMUNDO HERMÍNIO DE MELO.pdf]

Governo do Estado do Acre
Secretaria Estadual de Saúde
LABORATORIO CENTRAL DE SAUDE PUBLICA
Travessa do Hemoacre, 165 - Vila Ivonete
CNPJ: 04.034.526/0021-97
Resp. Técnico: Isaias Santos de Mesquita - **CRF:** CRBM N° 2679
Site: http://www.lacen.ac.gov.br - **E-mail:** produtos.lacen@ac.gov.br
Telefone: (68)3228-5355 - **Fax:** (68)3228-2720

RELATÓRIO DE ENSAIOS

Nº130118000414

Nº Vigilância: 13051510

DADOS DO SOLICITANTE

Nome: DEPARTAMENTO DE VIGILANCIA EPIDEMIOLOGICA E AMBIENTAL (CNES: 6917291)
Município: RIO BRANCO / AC
Natureza: PÚBLICA **Origem:** VIGIAGUA

DADOS DA COLETA

Finalidade: VIGIAGUA
Motivo: POTABILIDADE
Local: ESCOLA RAIMUNDO ERMINIO DE MELO
Endereço: BR 364 KM 07 RUA FLAVIANO MELO N° 175 SANTA CECILIA
Município: RIO BRANCO / AC
Zona: RURAL **Latitude:** -10°00`52,3" **Longitude:** -06°74`30,1"
Procedência da Coleta: ESTABELECIMENTO DE ENSINO
Ponto da Coleta: POÇO
Responsável: MAX **Documento:** RG 145422 **Telefone:** (06)9203-8983

DADOS DA AMOSTRA

Tipo da Amostra: ÁGUA NÃO TRATADA **Apresentação:** 400 mL **Acondicionamento:** GELO RECICLADO
Data da Coleta: 15/05/2013 **Hora da Coleta:** 13h 50min **Chuva nas últimas 48hs:** NÃO

ANÁLISE DE CAMPO

Não informado pelo responsável da coleta.

RECEBIMENTO DA AMOSTRA

Data: 16/05/2013 **Hora:** 09h 52min **Entregue por:** MAX **Recebido por:** JOEL

RESULTADO DAS ANÁLISES

MICROBIOLÓGICA

Ensaio: COLIFORMES TOTAIS **Data Final Processamento:** 17/05/2013 15h 47min
Referência: PORTARIA Nº 2.914, DE 12 DE DEZEMBRO DE 2011 **Valor Ref.:**
Metodologia: Substrato Cromogênico/Enzimático SMEWW, 21ª Ed.9223B
Resultado: Presença
Conclusão: Não Se Aplica
*Conferido e liberado por **FRANCISCO CARLOS DE ABREU MENEZES(Biomédico CRBM-4N°780-PA)**, em 17/05/2013 16:16:11.*

Ensaio: ESCHERICHIA COLI **Data Final Processamento:** 17/05/2013 15h 48min
Referência: PORTARIA Nº 2.914, DE 12 DE DEZEMBRO DE 2011 **Valor Ref.:** Ausência em 100 mL
Metodologia: Substrato Cromogênico/Enzimático SMEWW, 21ª Ed.9223B
Resultado: Ausência
Conclusão: Satisfatório
*Conferido e liberado por **FRANCISCO CARLOS DE ABREU MENEZES(Biomédico CRBM-4N°780-PA)**, em 17/05/2013 16:16:02.*

Governo do Estado do Acre
Secretaria Estadual de Saúde
LABORATORIO CENTRAL DE SAUDE PUBLICA
Travessa do Hemoacre, 165 - Vila Ivonete
CNPJ: 04.034.526/0021-97
Resp. Técnico: Isaias Santos de Mesquita - **CRF:** CRBM N° 2679
Site: http://www.lacen.ac.gov.br - **E-mail:** produtos.lacen@ac.gov.br
Telefone: (68)3228-5355 - **Fax:** (68)3228-2720

RELATÓRIO DE ENSAIOS

Nº130118000414

Nº Vigilância: 13051510

ORGANOLÉPTICA

Ensaio: COR APARENTE **Data Final Processamento:** 16/05/2013 12h 04min
Referência: PORTARIA Nº 2.914, DE 12 DE DEZEMBRO DE 2011 **Valor Ref.:** VMP: 15 uH
Metodologia: Método Espectrofotométrico SMEWW, 21ª Ed. 2120C
Resultado: 0 uH
Conclusão: Satisfatório

*Conferido e liberado por **SANDRA MARIA MARTINS DE SOUZA(Quimica CRQ 14100456)**, em 16/05/2013 12:38:56.*

Ensaio: TURBIDEZ **Data Final Processamento:** 16/05/2013 11h 39min
Referência: PORTARIA Nº 2.914, DE 12 DE DEZEMBRO DE 2011 **Valor Ref.:** VMP: 5 uT
Metodologia: Método Nefelométrico SMEWW, 21ª Ed. 2130B
Resultado: 0 uT
Conclusão: Satisfatório

*Conferido e liberado por **SANDRA MARIA MARTINS DE SOUZA(Quimica CRQ 14100456)**, em 16/05/2013 12:38:56.*

CONCLUSÃO FINAL

SATISFATÓRIA

*Conferido e liberado por **FRANCISCO CARLOS DE ABREU MENEZES(Biomédico CRBM-4N°780-PA)**, em 17/05/2013 16:05:05.*

Notas:
1 - VMP: Valor Máximo Permitido | VR: Valor de Referência;
2 - LQM: Limite de Quantificação do Método | LDM: Limite de Detecção do Método;
3 - SAA: Sistema de Abastecimento de Água | SAC: Solução Alternativa Coletiva | SAI: Solução Alternativa Individual;
4 - SMEWW: Standard Methods for the Examination of Water & Wastewater | APHA: American Public Health Association | NBR: Norma Brasileira;
5 - São de responsabilidade do solicitante o plano amostral, os dados da coleta, a coleta, o acondicionamento, o transporte e análise de campo;
6 - O relatório não pode ser utilizado em publicidade, propaganda e/ou para fins comerciais. Os resultados referem-se única e exclusivamente à amostra encaminhada pelo solicitante;
7 - Considerar Ausência em 100mL como valor de referência para Coliformes Totais apenas para amostra de ETA coletada na saída do tratamento/pós desinfecção. Nos outros pontos de coleta não há valor de referência para Coliformes Totais..
